# Progress on the correlation between sepsis and gut microbiota: a narrative review

**DOI:** 10.3389/fmicb.2026.1862891

**Published:** 2026-06-03

**Authors:** Yaning Yuan, Jiawei Li, Cheng Yang

**Affiliations:** 1 Department of Obstetrics and Gynecology Outpatient Nursing, West China Second University Hospital, Sichuan University, Chengdu, China; 2 Key Laboratory of Birth Defects and Related Diseases of Women and Children (Sichuan University), Ministry of Education, Chengdu, China; 3 Department of Pediatric Outpatient Nursing, West China Second University Hospital, Sichuan University, Chengdu, China; 4 Department of Pediatric Intensive Care Unit Nursing, West China Second University Hospital, Sichuan University, Chengdu, China; 5 Department of Pediatric Intensive Care Unit Nursing, WCSUH-Tianfu·Sichuan Provincial Children’s Hospital, Meishan, China

**Keywords:** antimicrobial stewardship, gut microbiota, probiotics, sepsis, source control

## Abstract

**Background:**

Sepsis is a life-threatening organ dysfunction with high mortality. Gut microbiota plays a key role in sepsis pathogenesis, but evidence is often mixed.

**Methods:**

This narrative review is based on a structured literature search (PubMed, Web of Science, Cochrane; from database inception to March 15, 2025). Findings are stratified into clinical (human), preclinical (animal), and unsafe/unproven categories.

**Results:**

Sepsis and gut microbiota form a bidirectional vicious cycle. Sepsis induces dysbiosis via ischemia, inflammation, and broad-spectrum antibiotics – the strongest iatrogenic driver. Dysbiosis in turn amplifies organ dysfunction through gut-brain, gut-lung, gut-liver, gut-kidney, and gut-heart/spleen axes. Clinically, adequate source control and antibiotic de-escalation are mandatory before any microbiota-directed therapy. Microbiota-targeted interventions show promise but carry significant risks: live probiotics are contraindicated in septic shock, severe pancreatitis, and immunocompromised patients (PROPATRIA trial).

**Conclusion:**

This evidence-stratified review provides a clinical roadmap: prioritize source control and antibiotic stewardship; avoid live probiotics in high-risk sepsis. Future research requires large RCTs and personalized strategies.

## 1 Introduction

Sepsis is a life-threatening organ dysfunction caused by a dysregulated host response to infection (Singer et al., 2016). With high incidence, rapid progression, and costly treatment, it imposes a sustained global burden (Lou et al., 2023; Niu and Chen, 2021). Despite advances in anti-infective therapy, fluid resuscitation, and organ support, mortality remains high, prompting the search for new therapeutic targets (Lou et al., 2022; Wiersinga and van der Poll, 2022).

The human gut harbors a vast microbial community (bacteria, archaea, fungi, viruses, protists) whose genetic content far exceeds the human genome (Gu et al., 2023). Under healthy conditions, the gut microbiota maintains a dynamic symbiotic balance with the host, participating in nutrient metabolism, vitamin synthesis, biological antagonism, and immune regulation, while the host provides a suitable environment and nutrients (Lou et al., 2023; Niu and Chen, 2021). Numerous studies show that gut microbial disturbances are closely linked to sepsis initiation and progression; sepsis disrupts the microbiota and aggravates end-organ dysfunction, while common therapeutic measures (broad-spectrum antibiotics, sedatives, parenteral nutrition) worsen dysbiosis, creating a vicious cycle (Lou et al., 2022; Zhao et al., 2023).

Innovative contributions of this review: Compared with previous reviews, this article has four distinctive features:

(1) It integrates bidirectional sepsis-microbiota mechanisms and explicitly grades evidence as clinical, preclinical, or conceptual to guide bedside application.
(2) It expands the gut-organ axis framework to include the gut-kidney and gut-heart/spleen axes, which are frequently omitted.
(3) It critically appraises microbiota-targeted interventions (probiotics, synbiotics, FMT, postbiotics, SDD) with risk–benefit analysis, highlighting contraindications (e.g., PROPATRIA trial showing increased gut necrosis in severe acute pancreatitis).
(4) It places the discussion within the clinical realities of source control and antibiotic stewardship, emphasizing that microecological interventions are adjuncts, not substitutes, for these fundamental measures.

Literature search strategy: we searched PubMed, Web of Science, and Cochrane Library from inception to March 15, 2025, using terms including “sepsis,” “septic shock,” “gut microbiota,” “intestinal microbiome,” “gut-organ axis,” “source control,” and “intra-abdominal infection.” Inclusion criteria: clinical studies, animal experiments, mechanistic studies; case reports and non-microbiome studies were excluded. The final reference list comprises original references, four recently published guidelines (suggested by reviewers), and additional key clinical studies identified during revision.

Evidence grading: throughout this review, we classify each major claim based on study design: [Clinical] (supported by human studies), [Preclinical] (supported only by animal or *in vitro* studies), [Clinical/Preclinical] (supported by both human and animal evidence), and [Clinical warning] (proven harmful in humans).

## 2 Bidirectional interplay between sepsis and gut microbiota

Sepsis and the gut microbiota exhibit a close bidirectional interaction, often forming a self-reinforcing vicious cycle: sepsis-induced systemic inflammation disrupts gut microecology, while pre-existing or secondary dysbiosis worsens sepsis severity, organ damage, and prognosis (Lou et al., 2023; Zhao et al., 2023). Although this bidirectional relationship is increasingly recognized as a key pathophysiological axis, relevant studies remain scarce, and the specific mechanisms and clinical implications are still unclear (Lou et al., 2022).

[Clinical/Preclinical].

### 2.1 Effects of sepsis on gut microbiota

The severe systemic stress of sepsis alters the gut environment at multiple levels, inducing dysbiosis through the following main mechanisms:

(1) Intestinal ischemia and reperfusion injury: Early sepsis reduces effective circulating blood volume and causes microcirculatory dysfunction. The gut, highly sensitive to perfusion changes, is among the first organs affected. Reduced mucosal blood flow leads to tissue ischemia, hypoxia, and epithelial cell apoptosis/necrosis, damaging the physical barrier. Subsequent reperfusion exacerbates injury via oxidative stress. Additionally, ischemia increases intraluminal oxygen, disrupting the anaerobic environment and promoting facultative anaerobe overgrowth, which further destabilizes microbial balance (Li et al., 2024; Zhang et al., 2022).
(2) Effects of systemic inflammatory mediators: Large amounts of inflammatory cytokines (e.g., TNF-*α*, IL-1β) released during sepsis not only cause systemic inflammation but also directly act on intestinal epithelial cells, altering barrier function, metabolism, and microbial interactions. For instance, cytokines regulate mucin and antimicrobial peptide expression, thereby shaping the microbial colonization microenvironment and driving compositional changes (Ling et al., 2023; Pérez-Hernández et al., 2021).
(3) Iatrogenic factors – the dominant clinical driver: Sepsis management strategies themselves contribute to gut dysbiosis. Early empirical broad-spectrum antibiotics, though lifesaving, indiscriminately suppress commensals and pathogens, causing a sharp decline in microbial diversity. ICU studies show that gut microbiota diversity in septic patients decreases within days and may persist for months after discharge. [Clinical] Even single-dose surgical antibiotic prophylaxis increases the risk of Clostridioides difficile colonization and infection (Sartelli et al., 2019). Moreover, sedatives/opioids inhibit intestinal motility, vasoactive drugs worsen ischemia, and parenteral nutrition lacks fermentable fiber—all perpetuating profound dysbiosis (Lou et al., 2022; Liu et al., 2020).

### 2.2 Role of gut microbiota dysbiosis in promoting sepsis

Gut dysbiosis is not a passive consequence but an active driver of sepsis aggravation, primarily through barrier dysfunction, immune-metabolic disturbances, and amplified systemic inflammation (Niu and Chen, 2021).

[Clinical/Preclinical].

(1) Bacterial and endotoxin translocation: Dysbiosis often coincides with impaired intestinal barrier function, together promoting translocation of bacteria and their products. These pathogen-associated molecular patterns (PAMPs), notably lipopolysaccharide (LPS), bind to Toll-like receptor 4 (TLR4) on immune cells, activating downstream signaling and inducing a second, more intense inflammatory storm that aggravates organ injury (Zhang et al., 2022; Panpetch et al., 2020).
(2) Loss of immunomodulatory function: A healthy gut microbiota maintains host immune homeostasis through multiple mechanisms. In sepsis, depletion of short-chain fatty acid (SCFA)-producing bacteria leads to markedly reduced SCFA levels, depriving the host of an important endogenous anti-inflammatory regulator (Lei et al., 2025; Macpherson et al., 2020). Dysbiosis also impairs immune cell function (e.g., alveolar macrophages), increasing secondary infection risk. Furthermore, vitamin D deficiency combined with dysbiosis may accelerate immunosenescence and chronic disease risk (Lou et al., 2022; Ullah, 2025).
(3) Metabolic disturbances and altered inter-organ communication: Beyond immune regulation, gut microbiota are deeply involved in host metabolism. Dysbiosis reduces beneficial metabolites (e.g., SCFAs) and may allow accumulation of harmful metabolites (e.g., hydrogen sulfide, ammonia), affecting energy metabolism and the normal function of liver, heart, and kidneys. Moreover, gut microbiota communicate bidirectionally with distant organs via “gut-organ axes” (e.g., gut-lung, gut-liver), influencing organ function in disease states (Cheng et al., 2023; Shen et al., 2025; Zhou et al., 2021).

**Figure 1:**
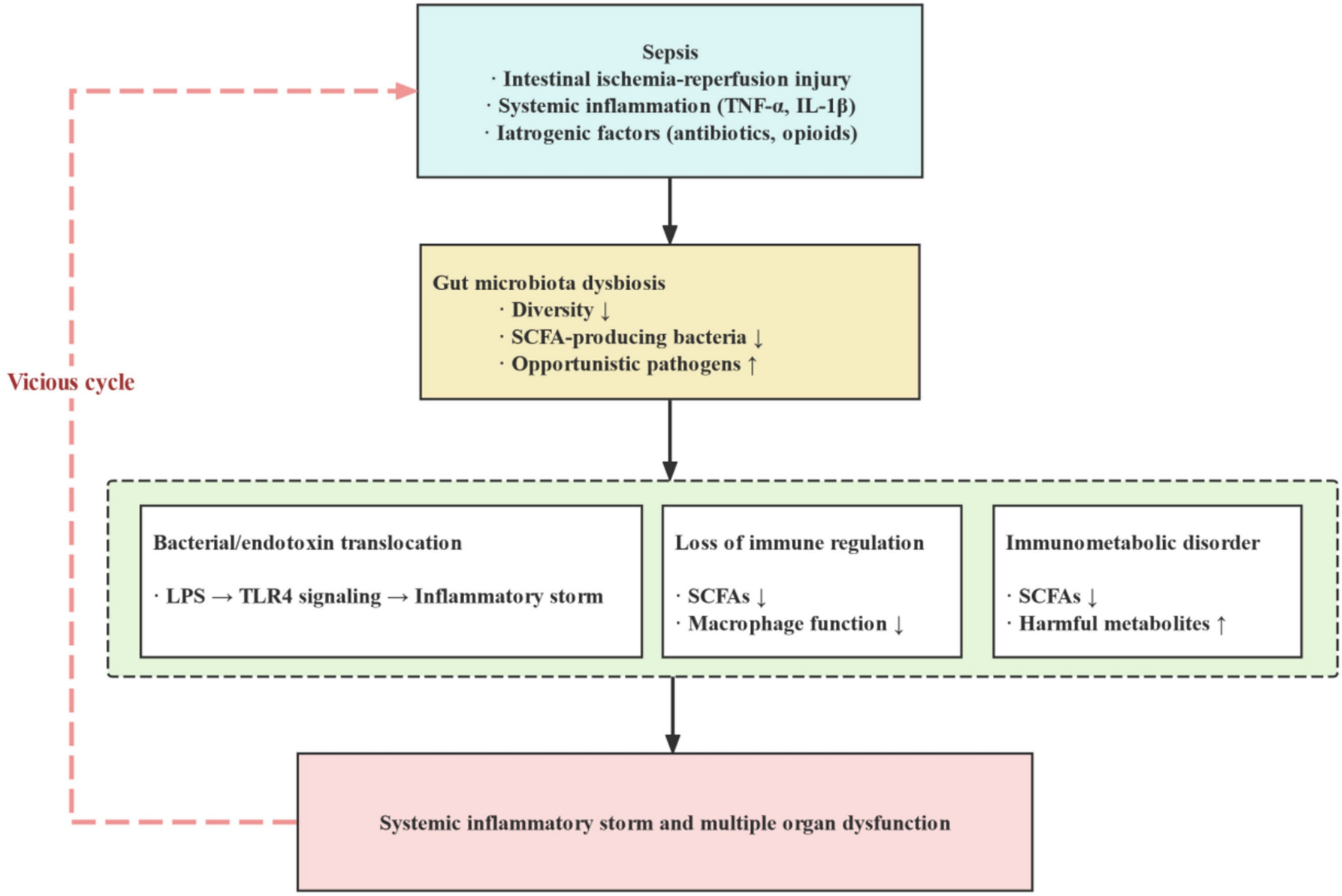
Bidirectional interplay between sepsis and gut microbiota. The schematic illustrates how sepsis triggers gut dysbiosis (left/upper part) and how dysbiosis subsequently amplifies sepsis severity (right/lower part), forming a self-reinforcing vicious cycle. Key mechanisms include bacterial translocation, immune dysregulation, and metabolic disturbances. SCFAs, short-chain fatty acids; LPS, lipopolysaccharide; TLR4, Toll-like receptor 4.

Summary: Sepsis and dysbiosis form a positive-feedback vicious cycle in which sepsis induces microbial disturbances, and these disturbances in turn promote multiple organ failure by amplifying inflammation, immune paralysis, and metabolic disorders. Understanding this cycle has important clinical implications for developing microecology-modulating strategies (e.g., probiotics, fecal microbiota transplantation, metabolite supplementation) (Widhani et al., 2022) (Figure 1).

## 3 Gut-organ axes: from gut dysbiosis to multiple organ dysfunction

**Figure 2:**
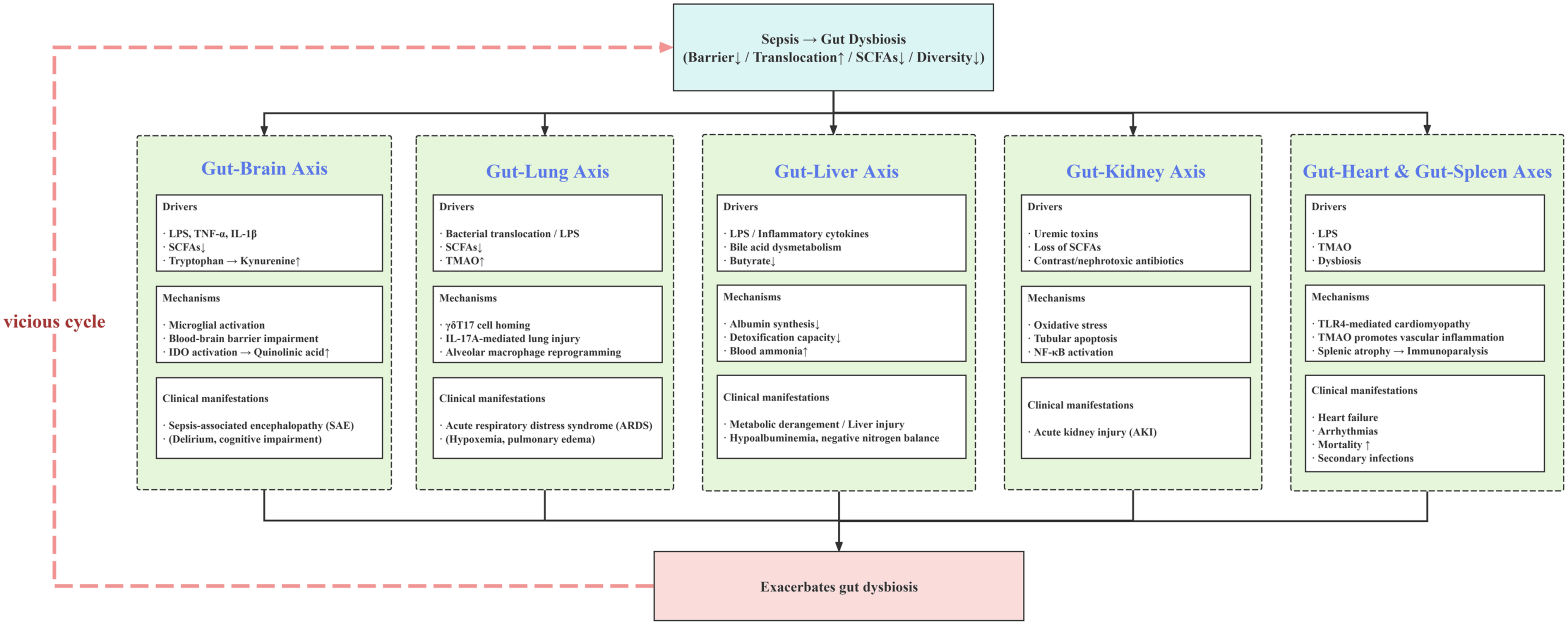
Schematic diagram of the gut-organ axes in sepsis. Sepsis induces gut dysbiosis, which subsequently drives organ dysfunction via multiple axes: gut-brain, gut-lung, gut-liver, gut-kidney, and gut-heart/spleen axes (detailed mechanisms shown in the figure). A red dashed arrow indicates the self-reinforcing vicious cycle. SCFAs, short-chain fatty acids; LPS, lipopolysaccharide; TMAO, trimethylamine N-oxide; SAE, sepsis-associated encephalopathy; ARDS, acute respiratory distress syndrome; AKI, acute kidney injury.

The clinical manifestations of sepsis reflect multi-organ dysfunction. Through “gut-organ axis” pathways, gut microbiota directly contribute to the development and severity of specific symptoms. Recent advances have revealed that gut microbiota not only influence sepsis via immune and metabolic routes but also through epigenetic regulation, mitochondrial function modulation, and neuroendocrine pathways (Niu and Chen, 2021; Giridharan et al., 2022). In addition to the well-recognized gut-brain, gut-lung, and gut-liver axes, emerging evidence highlights the gut-kidney and gut-heart/spleen axes (de Smet et al., 2009) (Figure 2).

### 3.1 Gut-brain axis and consciousness disturbance (SAE)

Sepsis-associated encephalopathy (SAE) occurs in 50–70% of septic patients, presenting as inattention, disorientation, agitation, and lethargy, and is a major cause of ICU delirium. SAE increases mortality, prolongs hospital stay, and may cause long-term cognitive dysfunction (Wang et al., 2024). The gut-brain axis – a bidirectional neural, endocrine, immune, and metabolic communication network – provides a mechanistic framework for SAE (Chang et al., 2024; O'Riordan et al., 2025).

[Clinical].

(1) Neuroinflammation: Dysbiosis and barrier disruption allow PAMPs (e.g., LPS) to enter the circulation, activate TLR4, and promote TNF-*α*, IL-1β, and IL-6 release. These cytokines enter the brain via circumventricular organs with a leaky blood–brain barrier (BBB), activate cerebrovascular endothelium, and increase BBB permeability (He et al., 2023). Germ-free mice have higher BBB permeability, which is restored by normal microbiota, indicating that gut microbes regulate BBB integrity. In sepsis, dysbiosis weakens this protection, allowing inflammatory mediators to enter brain parenchyma, damage neurons, impair synapses, activate microglia, and drive neuroinflammation (Lei et al., 2025; Sun et al., 2023).
(2) Microbial metabolites regulating neural function: SCFAs have systemic anti-inflammatory effects and can access the CNS or influence microglia via the vagus nerve. Reduced SCFAs in sepsis may shift microglia toward a pro-inflammatory phenotype, worsening neural injury (Gao et al., 2020; Liu et al., 2020). Many gut bacteria synthesize or modulate neurotransmitters (e.g., GABA by Lactobacillus/ Bifidobacterium; norepinephrine by *E. coli*, Bacillus, yeasts), potentially contributing to delirium and cognitive impairment (Sun et al., 2023; Yu and Zhao, 2021).
(3) Tryptophan metabolism shift: Under physiological conditions, tryptophan is metabolized mainly via the kynurenine and serotonin pathways. Inflammation induces indoleamine 2,3-dioxygenase (IDO), diverting tryptophan toward the kynurenine pathway, producing neurotoxic metabolites (e.g., quinolinic acid) while reducing serotonin synthesis. Gut microbiota participate in tryptophan metabolism; in sepsis, microbial disturbances may weaken protective mechanisms, while systemic inflammation enhances IDO-mediated neurotoxicity, together promoting consciousness disturbance (Sun et al., 2023).

### 3.2 Gut-lung axis and dyspnea (ARDS)

Acute respiratory distress syndrome (ARDS) – diffuse alveolar damage with inflammatory pulmonary edema – manifests as progressive dyspnea, refractory hypoxemia, and reduced lung compliance. The gut-lung axis represents an interactive network through which gut microbiota remotely regulate pulmonary immunity and inflammation (Chen et al., 2023; Xie et al., 2024).

[Clinical/Preclinical].

(1) Immune cell homing: Dysbiosis-generated local inflammatory signals activate gut mucosal immune cells, which then enter the circulation and home to the lung, where they release cytokines, recruit neutrophils, and aggravate lung injury – a “gut-primed, lung-manifested” pattern (Ziaka and Exadaktylos, 2024). Sepsis-induced small intestinal γδT17 cells migrate to the lung and trigger IL-17A-dependent injury. Gut microbiota composition also influences alveolar macrophage epigenetics; butyrate, for example, remodels macrophage inflammatory responses by inhibiting histone deacetylase, affecting ARDS severity (Xie et al., 2024; Ziaka and Exadaktylos, 2024).
(2) Systemic immunomodulation by microbial metabolites: SCFAs are key gut-lung axis signaling molecules. SCFA deficiency in sepsis may deprive the lung of anti-inflammatory regulation, permitting uncontrolled inflammation (Rastogi et al., 2022). Primary bile acids are converted by microbiota to secondary bile acids, which attenuate endotoxin-induced lung injury via the farnesoid X receptor (FXR) on lung epithelium. Plasma trimethylamine N-oxide (TMAO) levels correlate with oxygenation index and 28-day mortality in septic ARDS patients, potentially through TMAO-induced alveolar epithelial apoptosis and cytokine release (Ren et al., 2024; Tashiro and Shore, 2021).
(3) Direct translocation and ventilator-associated injury: Severe intestinal barrier failure allows gut bacteria and their products (e.g., LPS) to reach the lung via the portal or lymphatic circulation, directly activating alveolar macrophages and endothelial cells, triggering intense inflammation, increasing capillary permeability, and causing pulmonary edema. Mechanical ventilation itself can injure the lung, and dysbiosis further amplifies this injury, creating a mutually reinforcing vicious cycle between the gut and lung (Jin et al., 2020; Ma et al., 2025).

### 3.3 Gut-liver axis and metabolic derangement

The liver receives 70% of its blood from the gut via the portal vein, making it the primary organ for metabolism and immune regulation of gut-derived products (Xing et al., 2025). Sepsis-associated liver injury often presents as jaundice, coagulopathy, and metabolic disturbances, while nutritional disorders (muscle wasting, hypoalbuminemia, abnormal glucose/lipid metabolism, negative nitrogen balance) are common and closely linked to gut-liver axis impairment (Zhang et al., 2022; Sun et al., 2020).

[Clinical].

(1) Hepatic metabolic dysfunction: Bile acids are synthesized in the liver, released into the intestine, modified by microbiota, and reabsorbed – a cycle essential for fat and fat-soluble vitamin absorption (Zhra et al., 2025). In sepsis, dysbiosis disrupts bile acid metabolism, causing malabsorption and directly damaging hepatocytes and cholangiocytes, promoting steatosis and dysfunction (Hu et al., 2026; Mainali et al., 2021). Gut microbiota also participate in amino acid metabolism; in sepsis, high systemic protein catabolism leads to amino acid consumption by microbes or conversion into harmful products (ammonia, hydrogen sulfide, indole), exacerbating negative nitrogen balance and metabolic disturbances. Sepsis-induced dysbiosis dysregulates hepatic gluconeogenesis and lipid metabolism genes (Mainali et al., 2021; Yang et al., 2024).
(2) Impaired protein synthesis: Hypoalbuminemia in sepsis reflects not only vascular leakage but, more importantly, impaired hepatic synthesis. LPS and inflammatory cytokines inhibit albumin gene expression, while intestinal malabsorption limits amino acid supply, and hyperinflammation promotes muscle catabolism, all contributing to refractory hypoalbuminemia (Pérez-Hernández et al., 2021; Yang et al., 2024). Intestinal mucosal ischemia, edema, and villous shedding further reduce nutrient absorption. Butyrate promotes protein synthesis in enterocytes and hepatocytes; reduced butyrate in sepsis may impair this pathway, worsening hypoalbuminemia and muscle wasting (Jaeger et al., 2024).
(3) Impaired hepatic detoxification: The liver clears gut-derived toxins (ammonia, endotoxins, indole, phenols). In sepsis-associated liver injury, detoxification capacity declines, leading to systemic toxin accumulation (Oh et al., 2022). Hyperammonemia worsens consciousness disturbance; endotoxemia perpetuates systemic inflammation; and toxic metabolites suppress appetite and interfere with cellular metabolism, aggravating overall nutritional and metabolic disorders. Certain gut bacterial metabolites regulate hepatic cytochrome P450 enzymes, affecting drug metabolism and toxin clearance; dysbiosis may disrupt these detoxifying systems (Yu et al., 2022).

### 3.4 Gut-kidney axis and acute kidney injury (AKI)

[Clinical/Preclinical] Sepsis-associated AKI occurs in up to 50% of septic patients and independently increases mortality (Wang et al., 2025). The gut-kidney axis is a critical mediator: dysbiosis and increased permeability facilitate translocation of bacterial products and systemic accumulation of gut-derived uremic toxins, which directly damage renal tubules and exacerbate inflammation.

(1) Uremic toxins from gut microbiota: Gut bacteria metabolize dietary amino acids (tyrosine, phenylalanine, tryptophan) into indoxyl sulfate and p-cresyl sulfate. In sepsis, barrier impairment elevates circulating levels of these toxins. [Preclinical] Animal studies show indoxyl sulfate induces oxidative stress, proximal tubular apoptosis, and interstitial fibrosis (Wang et al., 2025). [Clinical] Higher indoxyl sulfate levels correlate with more severe AKI and prolonged renal replacement therapy (Wang et al., 2025).
(2) Reduced SCFAs: Loss of butyrate-producing commensals (e.g., *Faecalibacterium prausnitzii*, Roseburia spp.) during sepsis reduces SCFA availability. [Preclinical] Butyrate preserves renal function by inhibiting histone deacetylases, suppressing NF-κB activation, and reducing tubular epithelial apoptosis (Wang et al., 2025). [Clinical] Fecal SCFA levels are significantly lower in septic patients who develop AKI than in those without AKI (Wang et al., 2025).
(3) Iatrogenic renal insults that intersect with gut dysbiosis: [Clinical] In septic patients, contrast-enhanced imaging (e.g., CT for source identification) and nephrotoxic antibiotics (e.g., aminoglycosides, vancomycin) can compound AKI. These interventions further alter gut microbiota composition, creating a vicious cycle: contrast agents and antibiotics exacerbate dysbiosis, which in turn promotes renal injury via the mechanisms above (Wang et al., 2025).

Clinical implication: Strategies that preserve gut barrier function and restore SCFA production (e.g., early enteral nutrition with fiber, selective avoidance of unnecessary nephrotoxins) may reduce AKI severity, but this remains to be tested in prospective trials.

### 3.5 Gut-heart and gut-spleen axes

[Clinical/Preclinical] The heart and spleen are profoundly affected by gut-derived mediators during sepsis. The gut-heart axis contributes to sepsis-induced cardiomyopathy (SICM), while the gut-spleen axis drives immunoparalysis and secondary infections.

(1) Gut-heart axis and sepsis-induced cardiomyopathy: [Preclinical] Gut-derived LPS activates TLR4 on cardiomyocytes, leading to release of TNF-*α*, IL-1β, and IL-6, which directly suppress myocardial contractility. Selective gut decontamination attenuates cardiac dysfunction in animal models (Rastogi et al., 2022; Ren et al., 2024). [Clinical] Circulating gut-derived LPS levels are elevated in septic patients with reduced left ventricular ejection fraction and correlate with troponin elevation (Wang et al., 2025). Trimethylamine-N-oxide (TMAO): Gut microbiota metabolize dietary choline and carnitine to TMAO. [Clinical] High plasma TMAO is independently associated with increased risk of heart failure, arrhythmias, and 28-day mortality in septic patients (Rastogi et al., 2022; Wang et al., 2025). TMAO promotes foam cell formation, platelet hyperreactivity, and vascular inflammation.
(2) Gut-spleen axis and immunoparalysis: The spleen is a major reservoir of monocytes and B cells. [Preclinical] In septic mice, gut dysbiosis leads to splenic atrophy, loss of marginal zone B cells, and decreased production of IgM and opsonizing antibodies. [Clinical] Autopsy studies of septic patients show profound splenic lymphoid depletion, and low splenic volume on CT is associated with a higher risk of secondary nosocomial infections (Wang et al., 2025). The gut-spleen axis is bidirectional: splenic dysfunction further impairs gut mucosal immunity, perpetuating dysbiosis and translocation.

Clinical implication: Monitoring TMAO levels may help risk-stratify septic patients for cardiac complications. Preserving gut barrier integrity and restoring SCFA production could protect both heart and spleen, though this remains investigational.

## 4 Clinical integration: source control and antibiotic stewardship

Gut dysbiosis worsens sepsis, but two fundamental bedside measures must precede any microbiota-directed therapy: source control and rational antibiotic use. Failure to prioritize these has led to overoptimistic claims about probiotics and FMT.

### 4.1 Source control in intra-abdominal sepsis

[Clinical] Intra-abdominal infections (IAIs) are the second most common cause of sepsis. Source control (SC) is defined as measures to eliminate the infectious focus and restore homeostasis (Coccolini et al., 2023). Delay in adequate SC directly increases mortality; each hour delay in upper GI perforation raises mortality by 6% (Coccolini et al., 2023).

SC is not binary. The WSES/GAIS/SIS-E/SIS-A guidelines classify patients into three categories to guide aggressiveness (Coccolini et al., 2023):

- Class A: Healthy, infection is the main problem → standard surgery.
- Class B: Comorbidities but stable → cautious surgery ± stoma.
- Class C: Severe immunocompromise or instability → damage control (stoma, open abdomen).

Clinical implication: Class C patients are at highest risk of dysbiosis but least suitable for live probiotics due to bacteremia risk.

For specific IAIs (appendicitis, diverticulitis, etc.), the WSES/GAIS global clinical pathways provide detailed algorithms (Sartelli et al., 2021). Example – acute left colonic diverticulitis: uncomplicated cases may not need antibiotics; small abscesses (<4–5 cm) can be treated with antibiotics alone; larger abscesses require percutaneous drainage; diffuse peritonitis may need Hartmann’s procedure or primary anastomosis depending on stability (Sartelli et al., 2021).

### 4.2 Antibiotics: the double-edged sword

[Clinical] Broad-spectrum antibiotics are lifesaving but are the single most important iatrogenic cause of gut dysbiosis. They deplete colonization resistance, enabling overgrowth of Clostridioides difficile and multidrug-resistant organisms (MDROs) (Sartelli et al., 2019).

- CDI risk increases up to sixfold during and after antibiotic therapy; even single-dose surgical prophylaxis can trigger CDI (Sartelli et al., 2019).
- Duration: With adequate source control, 4 days of antibiotics is non-inferior to longer courses for complicated IAIs (Sartelli et al., 2021).
- De-escalation: Daily reassessment, using PCT where available, is essential to limit dysbiosis and resistance.

Take-home for clinicians: Before considering probiotics or FMT, ensure (1) source control is achieved, (2) antibiotics are narrowed/de-escalated as soon as possible, and (3) unnecessary PPIs are discontinued (PPIs increase CDI risk) (Sartelli et al., 2019).

## 5 Microbiota-targeted intervention strategies

Gut microbiota play an important role in sepsis, suggesting that targeting them could be therapeutic. Currently explored interventions include probiotics, synbiotics, fecal microbiota transplantation (FMT), postbiotics, and selective digestive decontamination (SDD). Below we summarize the evidence, distinguishing clinical benefit, uncertainty, and potential harm (Table 1).

**Table 1:** Summary of microecological intervention strategies in sepsis.

Intervention type	Representative preparations/Methods	Main mechanisms	Potential benefit	Uncertain benefit	Potential harm/Clinical warning
Probiotics	*L. rhamnosus GG, Bifidobacterium, S. boulardii*	Supplement beneficial strains	May reduce VAP and diarrhea in non-septic, immunocompetent ICU patients ([Clinical])	Efficacy in septic patients unproven	[WARNING] Bacteremia/fungemia; PROPATRIA trial: increased gut necrosis and mortality in severe acute pancreatitis. Do NOT use in septic shock, pancreatitis, or immunocompromised ([Clinical warning])
Synbiotics	Probiotics + FOS	Synergistic enhancement	Theoretical advantage over probiotics alone	No RCTs in sepsis ([Limited clinical])	Lower risk than probiotics alone? Unknown
FMT	Whole microbiota from healthy donor	Most thorough microbial reconstruction	[Established for recurrent CDI] Cure rate ~90% ([Clinical])	For sepsis: only preclinical evidence ([Preclinical])	[WARNING] Potential MDRO transmission, bacteremia, aspiration. Only in clinical trials for sepsis
Postbiotics	SCFAs (butyrate, propionate), tryptophan metabolites	Directly supplement beneficial metabolites	Avoids live bacteria risks; rapid onset	Only animal studies; no human trials ([Preclinical])	Theoretical risk of metabolic overload, but none reported
SDD	Oral tobramycin + polymyxin E + amphotericin B	Suppress pathogens, preserve anaerobes	May reduce CDI in some ICU settings	Inconclusive mortality benefit; risk of resistance ([Clinical – contradictory])	Long-term diversity loss; not recommended for routine sepsis care

FMT, fecal microbiota transplantation; FOS, fructooligosaccharides; VAP, ventilator-associated pneumonia; CDI, Clostridioides difficile infection; SCFAs, short-chain fatty acids; MDROs, multidrug-resistant organisms; PROPATRIA trial, a randomized controlled trial in severe acute pancreatitis that reported increased gut necrosis and mortality with probiotics (Besselink et al., 2008).

### 5.1 Probiotics

[Clinical] (for harm warning); [Clinical/Preclinical] (for potential benefit)

- Probiotics (Lactobacillus, Bifidobacterium, Saccharomyces boulardii) have been studied for preventing ventilator-associated pneumonia (VAP) and modulating intestinal barrier, but safety concerns are paramount in critically ill patients.
- Potential benefit: May reduce diarrhea and VAP in selected, immunocompetent, non-septic ICU patients.
- Potential harm: Cases of bacteremia/fungemia reported. Most importantly, the PROPATRIA trial in severe acute pancreatitis was stopped early because probiotics increased gut necrosis (9/152 vs. 0/144) and mortality (24/152 vs. 9/144) (Besselink et al., 2008). Live probiotics should NOT be routinely used in septic shock, severe acute pancreatitis, or immunocompromised patients.

### 5.2 Synbiotics

[Limited clinical] Probiotic–prebiotic combinations (e.g., with fructooligosaccharides) may enhance colonization resistance. Evidence in sepsis is limited to small studies; no harm signal yet, but also no robust efficacy.

### 5.3 Fecal microbiota transplantation (FMT)

[Clinical] (for recurrent CDI); [Preclinical] (for sepsis).

FMT is highly effective for recurrent *C. difficile* infection (cure rate ~90%) (Sartelli et al., 2019). For sepsis itself, only preclinical/animal studies exist (Han et al., 2024; Qian et al., 2025).

- Potential benefit: Restores microbial diversity and SCFA production in animal sepsis models.
- Potential harm: In critically ill patients, FMT carries risks of pathogen transmission (including MDROs), bacteremia, and aspiration. No RCT or prospective clinical study has evaluated FMT for sepsis unrelated to CDI; its use in this setting remains purely investigational and is not recommended outside of clinical trials.

### 5.4 Postbiotics

[Preclinical] Postbiotics (e.g., SCFAs, tryptophan metabolites) directly supply beneficial metabolites without live organisms, avoiding bacteremia risk. In animal sepsis models, SCFAs reduce mortality (Lou et al., 2022). Human trials are lacking, but this approach is conceptually attractive for critically ill patients.

### 5.5 Selective digestive decontamination (SDD)

[Clinical – contradictory] SDD uses oral non-absorbable antibiotics (tobramycin, polymyxin E, amphotericin B) to suppress potential pathogens while preserving anaerobes. Evidence from mixed ICU populations shows variable effects on CDI and mortality; long-term resistance and cost concerns limit routine use (Sartelli et al., 2021).

Major research limitations: Most microecological intervention studies are still preclinical or small-scale trials, with considerable heterogeneity. Challenges include high inter-individual variability, lack of standardized timing/strain/dosing, no consensus on an “ideal microbiota,” and potential risks – especially in immunocompromised patients (Han et al., 2024; Shang et al., 2024). Table 2 provides a systematic summary of key clinical studies (PROPATRIA trial Besselink et al., 2008; van Nood et al., 2013; Quraishi et al., 2017; de Smet et al., 2009; Widhani et al., 2022), detailing sample sizes, intervention protocols, primary endpoints, and safety data.

**Table 2:** Summary of key clinical studies on microbiota-targeted interventions in sepsis.

Intervention	Study (first author, year)	Study design	Sample size	Patient population	Intervention protocol	Key findings (primary endpoint included where applicable)	Safety/Adverse events
Probiotics	Besselink et al. (2008)	RCT, double-blind, placebo-controlled	296 (probiotics *n* = 152; placebo *n* = 144)	Predicted severe acute pancreatitis	Six probiotic strains (*L. acidophilus, L. casei, L. salivarius, L. lactis, B. bifidum, B. infantis*), twice daily for 28 days	Primary endpoint: infectious complications. Harm: Probiotics increased bowel ischemia (9/152 vs. 0/144, *p* = 0.004) and mortality (24/152 vs. 9/144). Trial stopped early.	Bowel ischemia leading to death
FMT	van Nood et al. (2013)	RCT, open-label	43	Recurrent Clostridioides difficile infection (non-sepsis)	Duodenal infusion of donor feces (*n* = 16) vs. vancomycin (*n* = 27)	Primary endpoint: resolution of diarrhea after 10 weeks. FMT cure rate 94% (15/16); vancomycin 31% (8/26).	No serious adverse events
FMT	Quraishi et al. (2017)	Systematic review + meta-analysis	37 studies (7 RCT, 30 case series)	Recurrent/refractory CDI	FMT via colonoscopy, nasogastric tube, or enema	Pooled resolution rate 92% (95% CI 89–94%). FMT superior to vancomycin (RR 0.23, 95% CI 0.07–0.80).	Serious adverse events uncommon (2.9%)
Synbiotics	Widhani et al. (2022)	RCT, double-blind, placebo-controlled	46 (*n* = 23 per group)	Systemic lupus erythematosus (SLE) – not sepsis	Synbiotics (probiotics + prebiotics) vs. placebo for 60 days	Primary endpoints: changes in gut microbiota, IL-6, hs-CRP. Synbiotics reduced IL-6 and stabilized hs-CRP; improved SLEDAI-2 K score.	No significant adverse events reported
SDD	de Smet et al. (2009)	Cluster randomized trial	5,939 ICU patients	Mixed ICU patients (not exclusively sepsis)	Oropharyngeal + digestive tract decontamination (tobramycin, polymyxin E, amphotericin B)	Primary endpoint: ICU mortality at day 28. SDD reduced ICU mortality (OR 0.83, 95% CI 0.72–0.97).	Long-term concerns for antimicrobial resistance

CDI, Clostridioides difficile infection; RCT, randomized controlled trial; ICU, intensive care unit; MDRO, multidrug-resistant organism; FMT, fecal microbiota transplantation; SDD, selective digestive decontamination; RR, relative risk; OR, odds ratio; CI, confidence interval; SLE, systemic lupus erythematosus; IL-6, interleukin-6; hs-CRP, high-sensitivity C-reactive protein; SLEDAI-2 K, Systemic Lupus Erythematosus Disease Activity Index 2000. The data in this table are derived from the following studies: PROPATRIA trial Besselink et al. (2008), van Nood et al. (2013), Quraishi et al. (2017), de Smet et al. (2009), and Widhani et al. (2022).

## 6 Key limitations and future directions of current research

Despite progress, significant challenges remain. We summarize the main limitations and future directions in Table 3.

**Table 3:** Key limitations and future directions in sepsis-gut microbiota research.

Domain	Main limitations	Recommended solutions	Priority research questions
Study design	Small sample size; predominantly cross-sectional, lacking longitudinal follow-up; inadequate control for confounders such as antibiotics	Large, multicenter RCTs; longitudinal designs; stratify by source control adequacy and patient class (A/B/C)	Dynamic changes of microbiota at different stages of sepsis; optimal timing for intervention
Microbiome analysis	Most studies use only 16S rRNA sequencing, lacking functional information; non-uniform pipelines	Multi-omics (metagenomics, metabolomics, proteomics)	Which functional pathways are most relevant to clinical outcomes? Reliable biomarkers?
Intervention strategies	Timing, dose, route not standardized; insufficient safety reporting	Precision microecological strategies tailored to baseline microbiota, severity, immune status	Which interventions for early (hyperinflammatory) vs. late (immune paralysis) stages?
Mechanistic studies	Insufficient understanding of specific molecular pathways	Deepen studies on receptor mechanisms, signal transduction, epigenetic regulation	Which strains or metabolites are key regulatory nodes?
Source control reporting	Most microbiome studies ignore SC adequacy	Adopt WSES/GAIS classification; require reporting of SC adequacy	Interaction between SC delay and dysbiosis severity?
Antibiotic stewardship	Antibiotic regimens, duration, de-escalation rarely reported	Mandate detailed antibiotic records; PCT-guided algorithms	Does shorter antibiotic duration preserve microbiome better without worsening outcomes?
Patient heterogeneity	One-size-fits-all approach ignores baseline microbiota and immune status	Precision therapy based on baseline microbiome (e.g., low SCFA producers vs. high Proteobacteria)	Develop rapid fecal SCFA or LPS assays to guide therapy.

RCTs, randomized controlled trials; SCFA, short-chain fatty acid; LPS, lipopolysaccharide; PCT, procalcitonin; WSES, World Society of Emergency Surgery; GAIS, Global Alliance for Infections in Surgery.

Specific recommendations for future research:

(1) Conduct large-scale, multicenter, rigorous RCTs to provide high-level clinical evidence.
(2) Integrate multi-omics (metagenomics, metatranscriptomics, metabolomics, proteomics) to characterize microbial and metabolite features across sepsis stages and subtypes, and identify reliable biomarkers.
(3) Develop individualized, precision microecological strategies tailored to baseline microbiota, disease severity, and immune status.
(4) Deepen mechanistic studies to elucidate specific molecular pathways (receptor mechanisms, signal transduction, epigenetic regulation) through which strains or metabolites modulate host responses via gut-organ axes, thereby identifying drug targets.
(5) Mandate reporting of source control adequacy and antibiotic de-escalation in all future microbiome studies in sepsis (Coccolini et al., 2023; Sartelli et al., 2021).

## 7 Conclusion

Sepsis is tightly linked to gut microbiota. This review examines the bidirectional interaction: sepsis induces dysbiosis through ischemia, inflammation, and iatrogenic factors, while dysbiosis aggravates sepsis and multi-organ dysfunction via barrier disruption, immune-metabolic disturbances, and gut-organ axes. This clinically oriented synthesis emphasizes:

(1) Sepsis and gut dysbiosis form a bidirectional vicious cycle driven by inflammation, ischemia, and broad-spectrum antibiotics – the strongest iatrogenic driver.
(2) Gut-organ axes (brain, lung, liver, kidney, heart, spleen) propagate local dysbiosis into systemic multi-organ dysfunction.
(3) Source control and antimicrobial stewardship are clinical non-negotiables that must precede any microbiota-directed therapy. Without adequate source control, microecological interventions are unlikely to succeed and may cause harm.
(4) Microbiota-targeted interventions (probiotics, FMT, postbiotics, SDD) show promise but carry serious risks in critically ill patients. Live probiotics are contraindicated in septic shock, severe acute pancreatitis, and immunocompromised states. Postbiotics (e.g., SCFAs) offer a safer, direct approach, but human data are lacking.
(5) Future research must adopt rigorous RCTs, multi-omics, and personalized stratification to translate these concepts into bedside therapies.

Final message to clinicians:

Before prescribing probiotics or considering FMT for a septic patient, ask:

- Is the source of infection controlled?
- Can antibiotics be de-escalated or stopped?
- Is the patient immunocompromised or in shock? If yes, avoid live probiotics.

Integrating basic microbiology with surgical and antimicrobial principles enables precision management of the gut–sepsis axis.
